# LHFFNet: A hybrid feature fusion method for lane detection

**DOI:** 10.1038/s41598-024-66913-1

**Published:** 2024-07-16

**Authors:** Youchen Kao, Shengbing Che, Sha Zhou, Shenyi Guo, Xu Zhang, Wanqin Wang

**Affiliations:** 1 School of Computer and Mathematics, Central South University of Forestry and Technology, Changsha, 410004 Hunan China; 2 School of Electronic Information and Physics, Central South University of Forestry and Technology, Changsha, 410004 Hunan China

**Keywords:** Engineering, Mechanical engineering

## Abstract

Lane line images have the essential attribute of large-scale variation and complex scene information, and the similarity between adjacent lane lines is high, which can easily cause classification errors. And remote lane lines are difficult to recognize due to visual angle changes in width. To address this issue, this paper proposes an effective lane detection framework, which is a hybrid feature fusion network that enhances multiple spatial features and distinguishes key features throughout the entire lane line segment. It enhances and fuses lane line features at multiscale to enhance the feature representation of lane line images, especially at the far end. Firstly, in order to enhance the correlation of multiscale lane features, a multi-head self attention is used to construct a multi-space attention enhancement module for feature enhancement in multispace. Secondly, a spatial separable convolutional branch is designed for the jumping layer structure connecting multiscale lane line features. While retaining feature information of different scales, important lane areas in multiscale feature information are emphasized through the allocation of spatial attention weights. Finally, considering that lane lines are elongated areas in the image, and the background information in the image is much more abundant than lane line information, the flexibility of traditional pooling operations in capturing widely existing anisotropic contexts in actual environments is limited. Therefore, before embedding feature output branches, strip pooling is introduced to refine the representation of lane line information and optimize model performance. The experimental results show that the accuracy on the TuSimple dataset reaches 96.84%, and the F1 score on the CULane dataset reaches 75.9%.

## Introduction

Researchers in the field have been drawn to the advancements in intelligent driving^1^. In the realm of intelligent driving, lane detection holds significant importance as it involves the utilization of the dedicated lane detection model to identify and locate the lanes on the road surface. This capability enables the vehicle to effectively navigate and align itself with the detected lane lines during driving. Accurate prediction of lane lines can help vehicles with more precise positioning and safer driving, which is a crucial prerequisite for intelligent driving.

**Figure 1:**
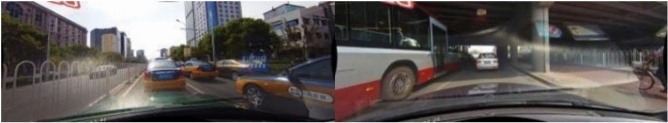
Lane line images in different scenarios.

Lane markings are designed according to specific patterns, primarily distinguished from the surrounding environment by their color^2,3^, shape^4,5^, and the meaning they represent. Therefore, early traditional lane detection methods mostly relied on manual feature extraction of low-level features such as color edges, and so on. However, traditional methods are unable to handle lane markings in different scenarios, as shown in Fig. 1, such as strong light and shadows, making it difficult to maintain robustness in real-world situations. Deep learning methods have demonstrated powerful capabilities in learning feature representations in complex scenes, thanks to the development of Convolutional Neural Networks (CNN). As a result, lane detection technology has made significant progress. Early methods based on deep learning detect lane lines through segmentation^6^. In recent years, various methods such as wavelet-based detection techniques^7,8^, anchor-based techniques^9,10^ and parameter prediction techniques^11^ have been proposed, continuously improving the accuracy and efficiency of lane detection algorithms. However, the limitations of CNN model are still apparent. There is evidence to show that CNN focusing on high and low-level semantic information and key local information can obtain more distinctive features^12^.

Research has shown that more distinctive features can enhance the recognition accuracy of network in complex scenes^13^. In addition, constructing multiscale CNN feature representations can overcome the limitation of narrow field of view caused by fixed input of CNN models^14^. The Stacked Hourglass Network (SHN)^15^ can connect features of different scales through jumping layers for feature extraction and fusion, in order to obtain lane features with resolution and semantic information. Ko^16^ et al. constructed a Point Instance Network (PINet) lane detection model using SHN as the backbone network and achieved excellent detection results. However, the construction of PINet has two drawbacks. Firstly, there is information redundancy, which makes it difficult to distinguish key features during the integration process. PINet fuses the upsampling of deep features with shallow features through jumping layers, which can lead to insufficient fusion and the redundant background information contained in deep features can interfere with accurate target localization. Secondly, PINet is simply constructed based on the structure of the backbone network. In actual lane line images, the width of lane lines may vary, and shallow extracted local features are insufficient to describe target features. Properly increasing the receptive field can obtain more detailed information and improve recognition performance.

In response to the above issues, this paper integrates multiscale local information and high-level and low-level global information to improve the recognition accuracy of existing CNN models. A Hybrid Feature Fusion Network for Lane (LHFFNet) with multi-space feature enhancement and multi-scale key feature differentiation is proposed to explore the impact of enhancing multiscale feature representation on improving the performance of network in detecting lane lines in complex scenes.

For different scene elements in lane line images, this paper enhances their feature representation through Multi-Head Self Attention mechanism (MHSA)^17^ after the output of each downsampling layer, enhances the correlation between adjacent layer features through inter layer transfer, and deepens the differentiation between different features to enhance the diversity of information in each layer. Thus, a Multi-space Attention Enhancement Module (MAEM) is proposed, which encodes the output of each sampling layer in multiple scales of features in multiple spaces. This can effectively enhance the output logic of subsequent feature fusion, making the output feature map more expressive. Considering the variability of spatial distribution in fused feature maps, a Space Concern Distinguish Module (SCDM) is developed to further emphasize key information regions in multi-scale feature maps. In addition, in order to eliminate the problem of inaccurate recognition of long-distance lane lines due to the decrease in lane pixel width caused by distance changes as much as possible, this paper introduces the Strip Pooling Module (SPM)^18^ structure to further refine the features before output. To investigate the feature extraction ability of this model for lane areas, this paper uses SHN as the backbone network and conducts experiments on the lane detection datasets TuSimple and CULane to test the overall performance of the model. The experimental results demonstrate that feature enhancement and key feature differentiation in the multi-space of the multi-scale features make the model more accurate in recognizing lane lines. In order to demonstrate the advancement of the proposed model, this paper compares the proposed LHFFNet with the current advanced methods, and obtains the best performance. The main contributions of this paper are summarized as follows:

- (1) To accurately encode the spatial information of each scale feature and carry out progressive feature enhancement, the MAEM method is proposed to enhance the output information of the last layer of each lower sampling layer in order to further capture spatial features.
- (2) As the spatial distribution of each scale feature will be different after the MHSA,the SCDM re-encodes the spatial information of each scale to further distinguish the key regions.
- (3) As the pixel width of the lane becomes narrower due to the change of the viewing angle at the far and near end of the lane, the SPM is introduced to flexibly obtain the lane marks at the far and near end, so that the lane can be identified more accurately.

## Related work

### Traffic line detection

A large portion of the previous techniques for lane detection relied on handcrafted low-level features to detect road markings^19,20^. These methods required complex feature extraction processes and were unable to effectively handle various complex scenarios due to the diversity of road scenes. Although some recent methods have addressed some limitations of traditional lane detection methods with more robust approaches, there is still room for improvement.

In recent times, deep learning has gained prominence as the prevailing method in computer vision research. According to the modeling methods of lane lines, current deep learning based lane line detection can be roughly divided into four categories: segmentation based methods, anchor based methods, keypoint based methods, and parameter prediction based methods. The segmentation based method treats the lane detection task as a pixel by pixel classification problem at the pixel level, with each pixel divided into two regions: lane lines and background. Usually, background pixel information far exceeds lane line information. In order to distinguish between lane lines, background information, and different types of lane lines, Pan et al.^6^ proposed Spatial CNN (SCNN), which treats lane line detection as a multi class segmentation task, treats different lane lines as different categories, and proposes a message passing mechanism to transfer information between adjacent rows or columns of the feature map. However, long-distance information dissemination can cause the loss of lane information, and the information transmission in SCNN is very time-consuming, resulting in slow inference speed. In response to the problem of low computational efficiency in SCNN, Zheng et al.^21^ proposed a Recurrent Feature-Shift Aggregator (RESA), which greatly improves computational efficiency through parallel information flow. Moreover, multiple step size information aggregation forms are more efficient and have lower losses compared to sequential aggregation in SCNN. Hou et al.^22^ applied the Self Attention Distillation (SAD) mechanism in ENet-SAD to capture global contextual information, achieving the aggregation and acquisition of rich contextual information by the network. Neven et al.^23^ constructed LaneNet, a multi task lane detection model using a branch structure, which includes two branches: binary segmentation and instance segmentation. Lane detection is considered as an instance segmentation problem, and clustering algorithms are used to assign corresponding lane line pixels to different lane lines to obtain each lane line.

The anchor based lane detection method first designs linear anchors, then calculates the offset between the sampling points and predefined anchors, and then uses Non Maximum Suppression (NMS) to filter out the lane lines with the highest confidence. Li et al.^1^ innovatively proposed a novel representation method for lane line anchors, which uses rays from different angles emitted from the left, bottom, and right boundaries of the image as anchors. Through this anchor, each lane line is divided into positive and negative samples, and the loss is calculated and the model parameters are updated. Qin et al.^7^ proposed a simple and efficient lane detection method, which transforms the lane detection task into finding a set of specific lane line positions in the image by selecting and classifying positions in the direction of the image. This method reduces the number of classifications, resulting in faster detection speed, but lacks robustness and generalization ability, resulting in average performance in complex scenarios.

Inspired by human pose estimation, some works consider lane detection tasks as key point estimation and correlation problems. Ko et al.^16^ proposed PINet for lane detection. This method uses the SHN as backbone network, based on keypoint estimation and instance segmentation methods to predict keypoint positions and embedded features, and uses clustering algorithms to classify different lane lines based on the similarity of embedded features. Qu et al.^24^ used a lightweight segmentation network that includes two branches, one for outputting heatmaps indicating whether pixels are keypoints, and the other for outputting offsets to precisely adjust the position of keypoints. Then, association algorithms were used to complete local to global curve correlation.

The method based on parameter prediction uses parameters to model lane lines and regresses these parameters to achieve the goal of lane line detection. Liu et al.^25^ proposed to output lane detection as model parameters related to the shape of lane lines, and established a lane line shape model based on road structure and camera posture, thereby providing reliable physical explanations for the parameters output by the network. This method uses the Transformer^17^ model to enhance feature expression and interaction in visual features, enabling the model to capture global contextual information in slender lane structures. This type of method relies on the accuracy of input parameters, and the instability of parameters can have a significant impact on lane modeling, leading to suboptimal detection performance.

### Feature representation enhancement

It is generally believed that the feature representation enhancement strategy should be universal for different types of images, including lane images, and many models use aggregate context information as one of the feature representation enhancement strategies. Feature representation enhancement is one of the main contents of this paper, and its related research is reviewed in this paper.

Jaderberg et al.^26^ proposed a model called spatial transformer network, which re encodes spatial information and enhances the representation of feature units to improve the detection performance of tasks such as image classification and object detection. SAGANs^27^ proposed a generative adversarial network model that utilizes self attention mechanism to aggregate contextual information in spatial images. This method significantly improves the quality of generated images by effectively combining self attention. Wang et al.^28^ proposed a method of using non local blocks to enhance the overall representation ability of the model for feature extraction units, providing a new approach for scene understanding. SCNN^6^ transforms traditional layer by layer convolution into layer by layer convolution in feature maps, thereby effectively extracting and utilizing spatial information across rows and columns in traffic scenes. However, this model requires a large amount of training resources for optimization, and information loss may occur in scenarios involving remote information transmission. RESA^21^ is a module used for aggregating spatial information, which captures local and global features through recursive feature aggregation, allowing features to be fully described. However, this structure requires computing and transmitting features at each layer, resulting in high computational complexity. Hou et al.^22^ applied self attention distillation (SAD) mechanism in Enet-SAD for global feature enhancement, achieving the aggregation and acquisition of rich contextual information in the network.

## Method

### Overall architecture

**Figure 2:**
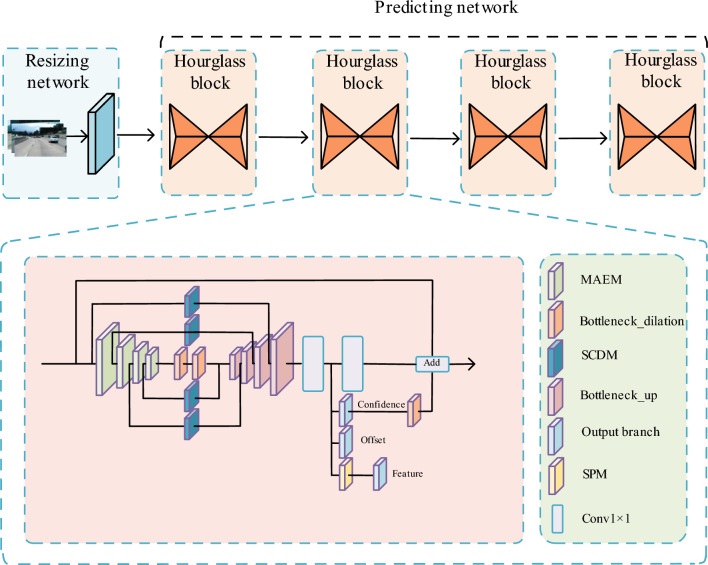
Overview of LHFFNet. LHFFNet consists of an resizing network and a predicting network. The prediction network consists of four hourglass blocks.

This section presents the LHFFNet framework, illustrated in Fig. 2. The network is divided into two parts: resizing network and predicting network.

In LHFFNet, there are four hourglass modules and one adjustment network. Firstly, MAEM is used to extract lane features while enhancing attention to feature information at various scales, enhancing feature representation, optimizing information transmission within the hourglass module, and ensuring that information is not blurred or lost during transmission across hourglass modules. Then, each hourglass module utilizes the SCDM to retain feature information at different scales. In the original jumping layer of the SHN, due to equal pixel weights, the original jumping layers in SHN cannot effectively distinguish between key and non key regions in the multiscale feature maps, which has a negative impact on the distinguishability of the feature extraction. To address this issue, this paper designs SCDM to emphasize the structure of key regions with high weights, enabling the network to balance feature maps with different weights, in order to better distinguish lane lines from the background, expand the output logic of MAEM, and make information at different scales more distinguishable. Finally, the SPM is used in the output branch to further refine the extraction of lane information, flexibly encoding the remote lane information in space, and improving the ability that model detects lane lines at the remote position.

Specifically, after the image is input into the network, the network is first resized for processing operations, reducing the size of the input image to save storage space and inference time, while also reducing computational complexity. Afterwards, the output of the resizing network will be input into the predicting network for further feature extraction, and the prediction results will be output. The MAEM enhances the feature extraction capability of the model. After using the residual module to extract features, the obtained feature map is enhanced by using MHSA for multi-space feature expression, guiding the network to obtain global contextual information and global semantic features, establishing strong correlation between feature information, and reducing information loss between hourglass modules. At the same time, the SCDM connects feature maps of different scales in the backbone network, and introduces new spatially separable convolution branches into the original jumping layer, giving each pixel region a separate weight. This can effectively emphasize the key areas around the lane features, enhance the importance of spatial features, and enhance the ability of model to learn spatial positions. Then, the output of MAEM is processed by distillation layers and fused with the output features of SCDM for upsampling. The outputs of each scale during the upsampling process are fused with the output results of SCDM for feature fusion. Subsequently, subsequent operations such as upsampling are performed to predict confidence, offset, and embedded features refined by SPM. Based on the output results, the points on the lane are predicted and then fitted.

### Multi-space attention enhancement module

**Figure 3:**
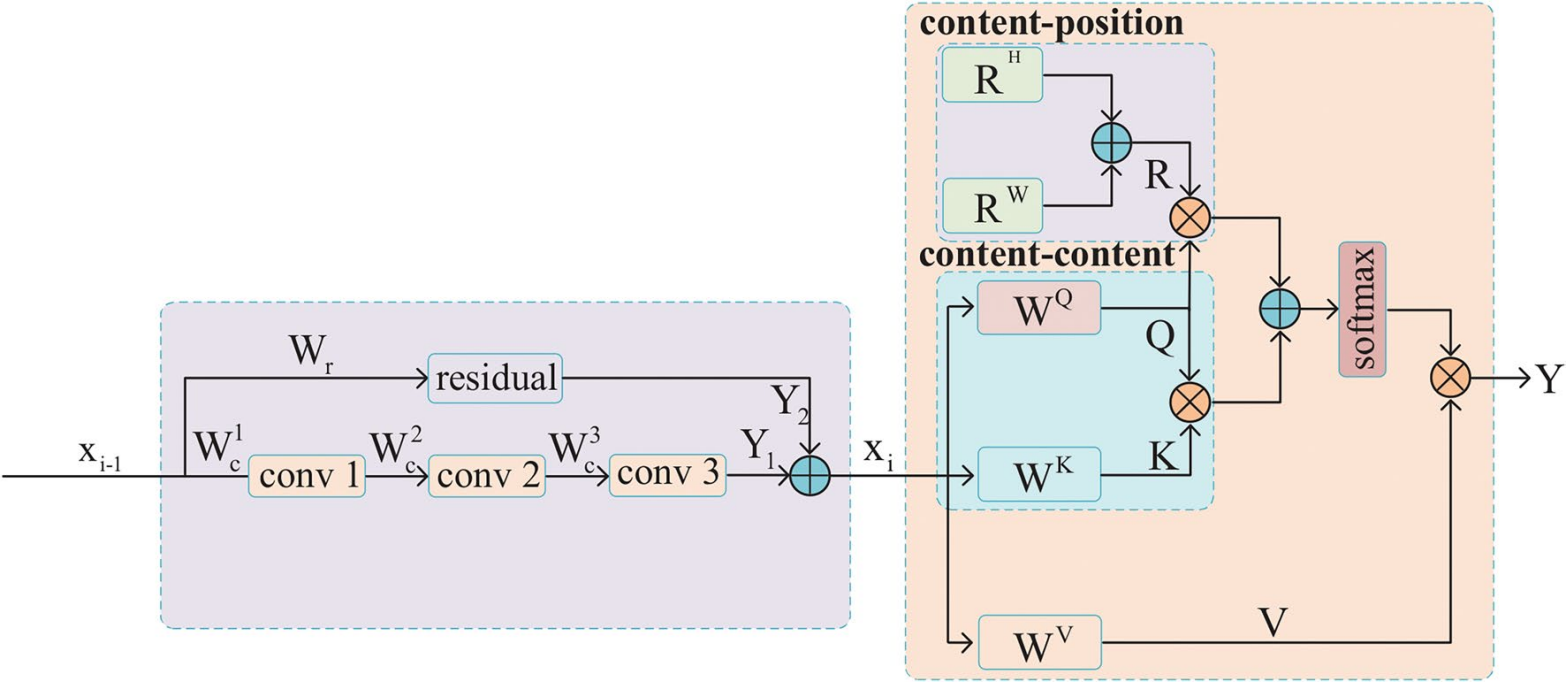
The structure of the MAEM. The first part is residual structure, and the second part is MHSA using relative position encoding.

Due to excessive noise in the lane lines images, the lane markings are not clear. In the process of extracting lane line features in the network, there are certain differences in the information extracted in each channel of the feature map at each scale. Conventional convolution operations can only extract limited local features of the lane, lacking a global perspective. As the network goes deeper, the interesting areas in the network gradually change from local textures to global contours, and the local semantic information contained in the shallow layer is equally important for determining the location of lane lines that accurately locate important information in the scene, so it also needs to be emphasized. MHSA is used for feature representation enhancement after residual structure in MAEM, so that both shallow local important information and deep global semantic information can be grasped by the model. The study by Srinivas et al.^29^ shows that the hybrid design mechanism of convolution and self attention enables the model to effectively capture local and global information and establish remote dependencies, collect and correlate scene information, obtain global features of data, enhance relationships between objects, and establish strong correlations between data. This provides strong theoretical support for the design of MAEM. The structure diagram of MAEM is shown in Fig. 3, which consists of two components.

The first component is a residual structure designed to extract feature information and generate feature maps of a specific size. The operation process can be expressed as:

1 $$\begin{array}{c}{Y}_{1}={conv}_{1\times 1}\left({conv}_{3\times 3}\left({conv}_{1\times 1}\left({x}_{i-1},{W}_{c}^{1}\right),{W}_{c}^{2}\right),{W}_{c}^{3}\right)\end{array}$$

2 $$\begin{array}{c}{Y}_{2}={conv}_{3\times 3}({x}_{i-1},{W}_{r}^{1})\end{array}$$

3 $$\begin{array}{c}{{x}_{i}={Y}_{1}+Y}_{2}\end{array}$$

where ${x}_{i-1}$ is the original input feature map, and ${W}_{c}^{1}$, ${W}_{c}^{2}$ and ${W}_{c}^{3}$ represent the parameters of the convolution branch and residual branch respectively. ${Y}_{1}$ and ${Y}_{2}$ represent the output of two branches. ${x}_{i}$ is the sum of the output of the two branches.

The second part is MHSA containing relative position encoding^30^. MHSA takes the output of last convolutional layer in the residual structure as input and transforms the input features using linear transformation matrices ${W}^{Q}$, ${W}^{K}$ and ${W}^{V}$ to obtain query, key and value. Relative position encoding is represented by ${R}^{H}$ and ${R}^{W}$ to improve the position perception of MHSA. Previous studies have shown that relative position encoding is beneficial for visual tasks^31,32^, as it allows attention to be associated with the relative distance between different positional features. Sum ${R}^{H}$ and ${R}^{W}$, and perform dot multiplication with the query to obtain the correlation score of the content position. Perform dot multiplication with the key and query to obtain the similarity score of the content. Sum the two scores and generate weights using the Softmax function. Finally, apply the weights to the value vectors corresponding to the feature map to generate attention. If the input feature map is represented as ${x}_{i}$, the attention generation process can be summarized as follows:

4 $$\begin{array}{c}Attention(Q,K,V,R)=(Softmax\left({Q}^{T}K+{Q}^{T}R\right){)}^{T}V\end{array}$$

where the query $Q={x}_{i}{W}^{Q}$, key $K={x}_{i}{W}^{K}$, value $V={x}_{i}{W}^{V}$ and $R={R}^{H}+{R}^{W}$.

The ${W}^{Q}$, ${W}^{K}$ and ${W}^{V}$ are parameter matrices that are independently learned for each attention head.They are used to perform linear transformations on the query, key and value. Instead of performing a single attention function, MHSA allows each attention to learn different feature representations. This increases the capacity and generalization of model by learning multiple features. The working principle of MHSA is to divide the input features into N groups and calculate single-head attention for each group using different transformation matrices and positional encodings. The attention mechanism is applied in parallel for each single-head attention calculation. The outputs are then concatenated and projected again to generate the final output, capturing information from different subspaces. The computation for each attention head can be written as:

5 $$\begin{array}{c}Hea{d}_{i}=Attention\left({x}_{i}^{i}{W}_{i}^{Q},{x}_{i}^{i}{W}_{i}^{K},{x}_{i}^{i}{W}_{i}^{V},{R}_{i}\right)\end{array}$$

where ${x}_{i}^{i}$ represents the i-th group obtained by dividing the input feature mapping into N groups, ${W}_{i}^{Q}$ , ${W}_{i}^{K}$ and ${W}_{i}^{V}$ are the linear transformation matrices for the i-th group, ${R}_{i}$ is the positional encoding for the i-th head, and $Hea{d}_{i}$ represents the attention of the i-th head.

After obtaining the attention weights for each spatial dimension, the formula for calculating the multi-space $Attention\left(Q,K,V,R\right)$ concatenation can be described as:

6 $$\begin{array}{c}MHSA\left(Q,K,V,R\right)=Concat\left(Hea{d}_{1},Hea{d}_{2},\dots ,Hea{d}_{n}\right){W}^{*}\end{array}$$

where ${W}^{*}$ is the matrix used for linear transformation, and $MHSA\left(Q,K,V,R\right)$ is the output of the MAEM.

### Space concern distinguish module

**Figure 4:**
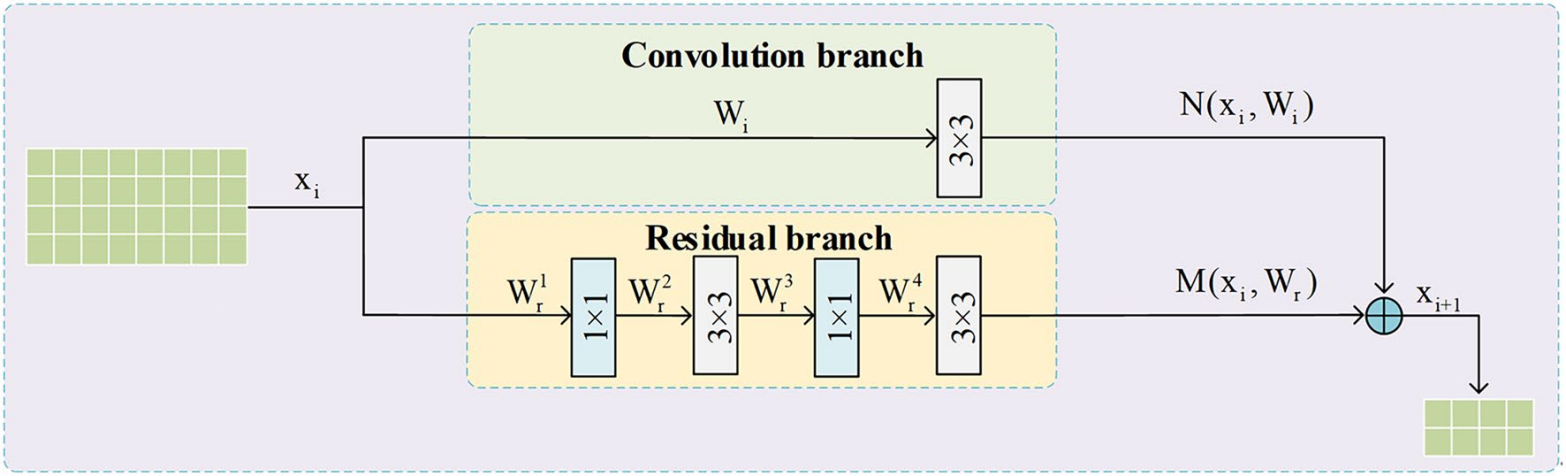
The original jumping layer structure of PINet. It only contains one convolution branch and one residual branch.

**Figure 5:**
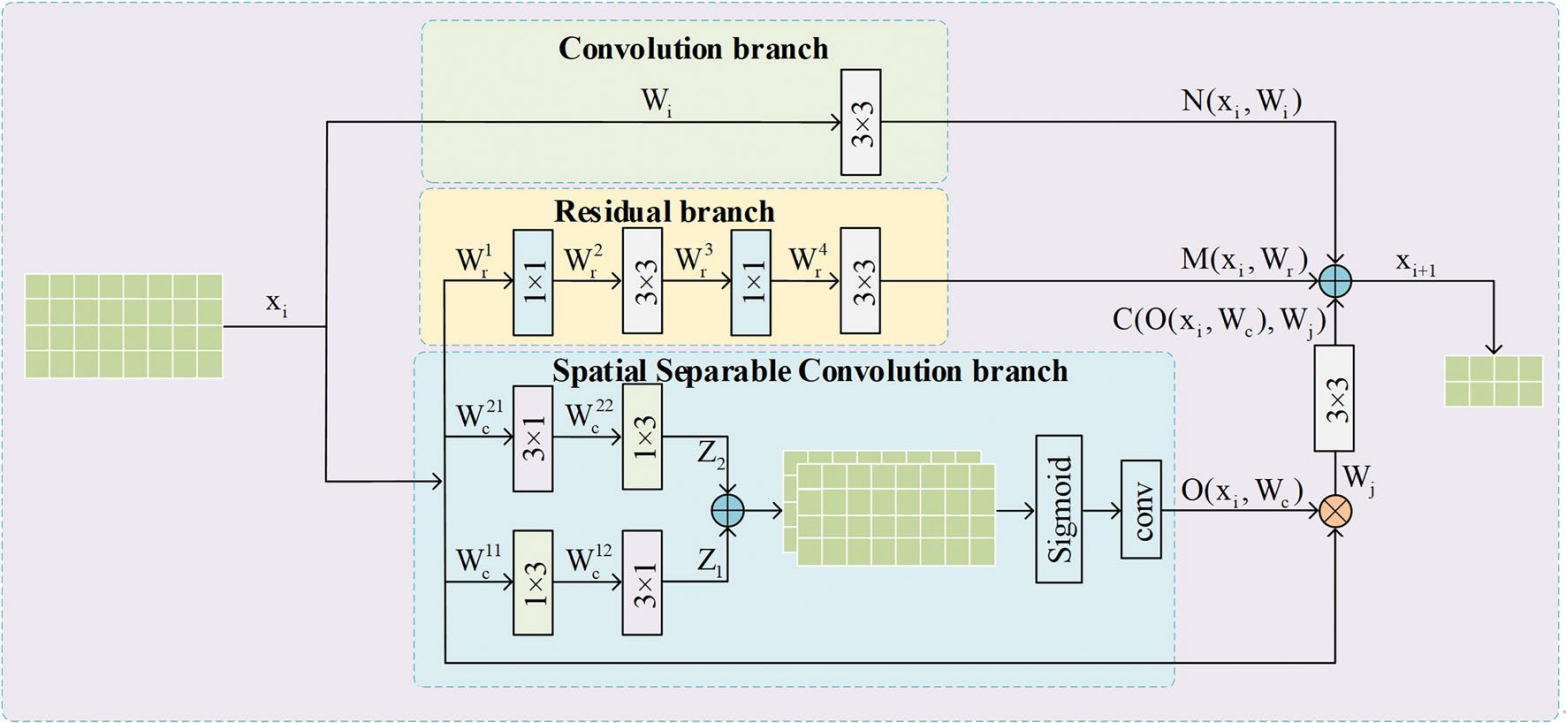
The structure of SCDM. Compared to Fig. 4, it adds spatial separable convolution branch to re encode multiscale information and filter important regions.

In PINet, the original jumping layer of SHN consists of a convolution branch and a residual branch. Its structure is given in Fig. 4. As discussed before, the two branches in the original jumping layer can integrate feature information from various scales, but they can not effectively emphasize the crucial regions around lane features. As a result, the original design of SHN cannot adequately recognize and extract lane information. In order to solve the the problem, a spatial separable convolution branch is introduced into the SCDM. The SCDM consists of three main parts. Its structure is given in Fig. 5. The first part is the regular convolutional branch, which aims to extract features and adjust the input feature map to an appropriate size for subsequent fusion operations. Its output can be described as follows:

7 $$\begin{array}{c}N\left({x}_{i},{W}_{i}\right)=con{v}_{3\times 3}\left({x}_{i},{W}_{i}\right)\end{array}$$

where ${x}_{i}$ represents the input feature map, ${W}_{i}$ is the parameters in the convolution branch.

The second part is the residual block branch, which consists of four convolutional operations. Its output can be represented as follows:

8 $$\begin{array}{c}M\left({x}_{i},{W}_{r}\right)=con{v}_{3\times 3}\left(con{v}_{1\times 1}\left(con{v}_{3\times 3}\left(con{v}_{1\times 1}\left({x}_{i},{W}_{r}^{1}\right),{W}_{r}^{2}\right),{W}_{r}^{3}\right),{W}_{r}^{4}\right)\end{array}$$

where ${W}_{r}=$ ${W}_{r}^{1}, {W}_{r}^{2}, {W}_{r}^{3}, {W}_{r}^{4}$ are the parameters in the residual branch.

The third part is the spatial separable convolution branch, inspired by Hou et al.^15^, the spatial separable convolution branch adopts a ”split-merge” framework structure, using consecutive 1 × 3 and 3 × 1 spatial separable convolutions to spatially encode multi-scale feature information. The original data is input into two branches respectively for convolution, and then pixel level fusion is carried out. The map after fusion is performed by the Sigmoid function to obtain the normalized result. Then, channel reduction operation is performed using convolution. This results in the final output feature map $O({x}_{i},{W}_{c})$. In fact, this branch can be seen an attention module, where the output feature map $O({x}_{i},{W}_{c})$ can be viewed as a weight map. The process of obtaining $O({x}_{i},{W}_{c})$ can be described as follows:

9 $$\begin{array}{c}{Z}_{1}=con{v}_{3\times 1}\left(con{v}_{1\times 3}\left({x}_{i},{W}_{c}^{11}\right),{W}_{c}^{12}\right)\end{array}$$

10 $$\begin{array}{c}{Z}_{2}=con{v}_{1\times 3}\left(con{v}_{3\times 1}\left({x}_{i},{W}_{c}^{21}\right),{W}_{c}^{22}\right)\end{array}$$

11 $$\begin{array}{c}O\left({x}_{i},{W}_{c}\right)=conv\left(\sigma \left({Z}_{1}+{Z}_{2}\right)\right)\end{array}$$

where ${W}_{c}={W}_{c}^{11}, {W}_{c}^{12}, {W}_{c}^{21}, {W}_{c}^{22}$ are the parameters in the spatial separable convolution branch, ${Z}_{1}$ and ${Z}_{2}$ are the outputs of two separble convolutions, $\sigma$ is the sigmoid function, and conv represents the convolutional block for channel reduction.

Then the obtained weight mapping is multiplied with the original input feature map, allowing each pixel region to have a separate weight. The purpose is to differentiate different regions on the original feature map and assign larger weights to key regionsduring subsequent backpropagation, thereby emphasizing the key areas. In SCDM, the output feature dimensions height and width for each ${x}_{i+1}$ are determined to be half of the original input feature map. As the size dimensions of $O({x}_{i},{W}_{c})$ are identical to those of the original feature map, another convolution operation is performed to adjust its dimensionality for the subsequent final fusion operation. The convolution process that reduces the size of height and width can be writeen as follows:

12 $$\begin{array}{c}C\left(O\left({x}_{i},{W}_{c}\right),{W}_{j}\right)=con{v}_{3\times 3}\left({x}_{i}O\left({x}_{i},{W}_{c}\right),{W}_{j}\right)\end{array}$$

where $C(O({x}_{i},{W}_{c}),{W}_{j})$ is the output feature map obtained by convolution, ${x}_{i}O({x}_{i},{W}_{c})$ is the result of fusing ${x}_{i}$ and the feature map obtained from spatial separable convolution through matrix multiplication, and ${W}_{j}$ represents the adjustable parameters of this convolution. The ${x}_{i+1}$ is obtained by fusing these three outputs, and this process can be described as follows:

13 $$\begin{array}{c}{x}_{i+1}=N\left({x}_{i},{W}_{i}\right)+M\left({x}_{i},{W}_{r}\right)+C\left(O\left({x}_{i},{W}_{c}\right),{W}_{j}\right)\end{array}$$

where ${x}_{i+1}$ is the final result after merging the three branch results, $N({x}_{i},{W}_{i})$, $M({x}_{i},{W}_{r})$ and $O({x}_{i},{W}_{c})$ are the output results of the convolution branch and residual branch and spatial separable convolution branch respectively. $C(O({x}_{i},{W}_{c}),{W}_{j})$ represents the result of matrix multiplication between $O({x}_{i},{W}_{c})$ and the original input feature map, followed by convolutional dimension reduction.

As shown in Eqs. (7)-(13), the SCDM is described in detail. Based on the ”split-merge” dual-branch structure, it uses spatial separable convolution to obtain weight maps, which are then fused with the original input feature map, the residual branch, and the output feature maps of the convolution branch. This fusion process not only extracts important features but also emphasizes key regions with higher weights, capturing both global lane features and paying more attention to various forms of lane information and the surrounding key regions in the feature map. Therefore, SCDM is beneficial for learning more discriminative features in the lane detection process.

### Strip pooling module

**Figure 6:**
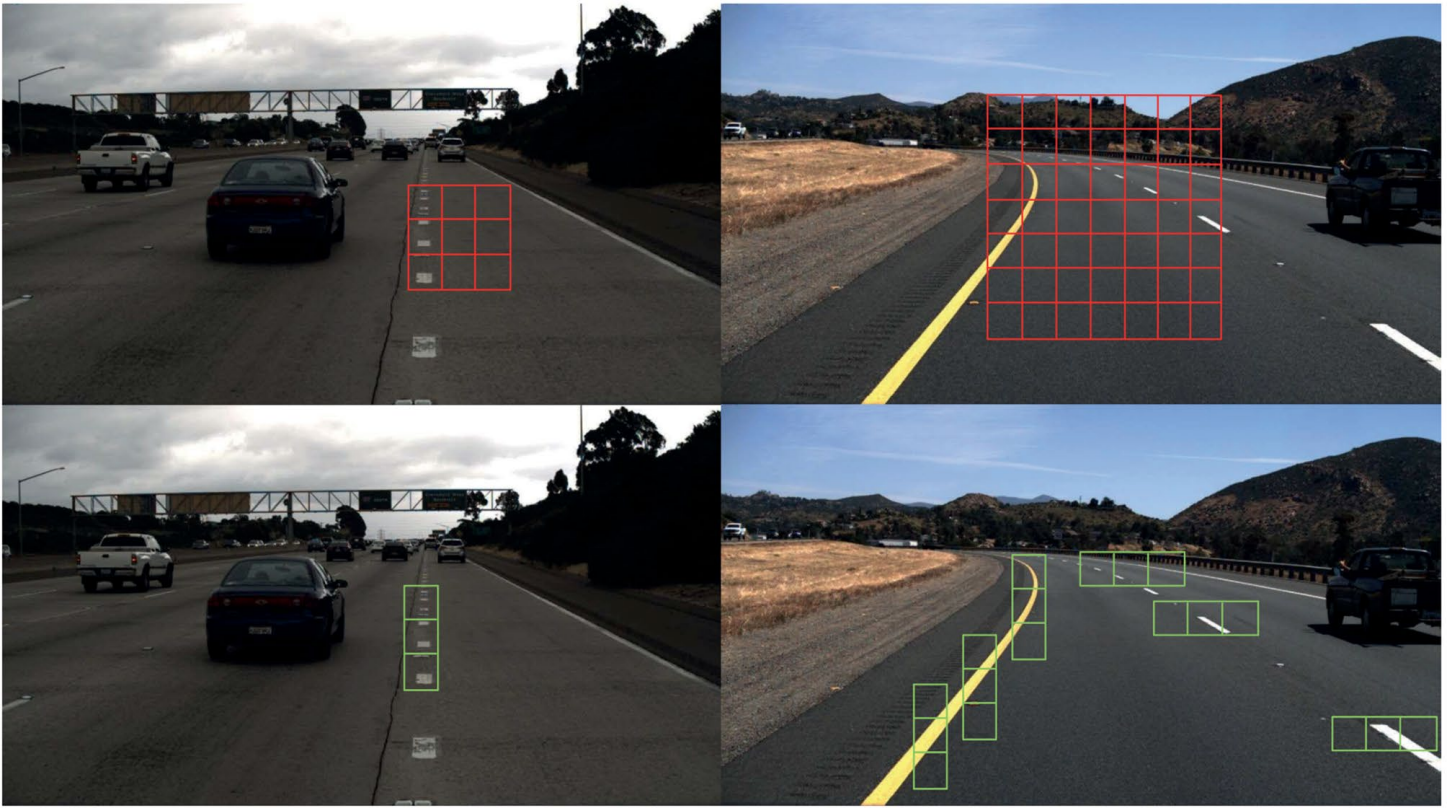
Strip pooling and spatial pooling have different effects on analyzing different scenes. Compared to traditional pooling, strip pooling (green grid) can better extract local information of the lane and capture long-range dependencies (horizontal green squares).

In instance segmentation tasks, achieving fine-grained segmentation of feature maps at different positions is highly challenging. The features of lane lines are usually distributed at different locations in an image and are often accompanied by occlusions and wear. Additionally, due to visual reasons, the width of lane lines can vary between near and far regions. Since lane lines themselves are long strips with straight or curved shapes that can appear in any local region of the image, it requires the model to pay more attention to these local details when segmenting the feature maps. Traditional average pooling methods may inevitably include many irrelevant regions when dealing with complex scenes containing lane lines and other objects. As shown in the red grid in Fig. 6, these methods are also limited in capturing the flexibility of anisotropic context that widely exists in real-world scenes, especially for long strip-like objects like lane lines.

**Figure 7:**
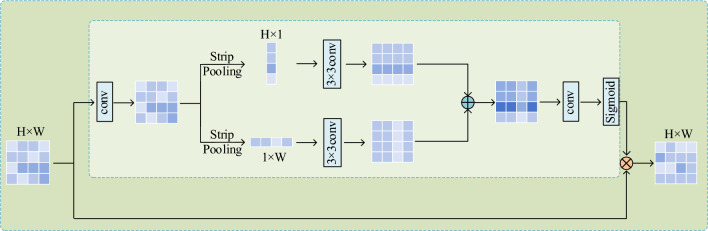
The structure of SPM.

Inspired by Hou et al.^18^, this paper introduces the SPM before the output branch of the hourglass block. Figure 7 shows the architecture of the SPM. Accurately extracting lane line features allows the model to enhance segmentation accuracy, leading to improved detection performance by precisely segmenting lane lines, particularly at remote edge positions.

The SPM introduces the concept of strip pooling. In strip pooling, a 2D tensor $x\in {R}^{H\times W}$ is subjected to spatial pooling along either the horizontal or vertical dimension. This pooling operation involves averaging the feature values in each row or column of the tensor. The resulting output after horizontal strip pooling, denoted as ${z}^{h}\in {R}^{H}$, while the output after vertical strip pooling, denoted as ${z}^{v}\in {R}^{W}$ . ${z}^{h}$ and ${z}^{v}$ can be expressed as:

14 $$\begin{array}{c}{z}_{m}^{h}=\frac{1}{W}\sum_{0\le n<W} {x}_{m,n}\end{array}$$

15 $$\begin{array}{c}{z}_{n}^{v}=\frac{1}{H}\sum_{0\le m<H} {x}_{m,n}\end{array}$$

The pooling layers with long and narrow kernel shapes play a crucial role in capturing long-range dependencies in sparsely distributed regions of lane lines and encoding regions with a banded shape. Simultaneously, due to their narrow core shape along the other dimension, they effectively capture local details. Moreover, strip pooling operations enable the collection of remote contexts from different spatial dimensions.

Assuming an input tensor $\in {R}^{C\times H\times W}$ , where *C* represents the number of channels. This tensor is first fed into two distinct branches, each consisting of a horizontal or vertical strip pooling layer. Subsequently, a 1D convolutional layer with a kernel size of 3 is applied to modulate the current location and its neighboring features. This process produces two intermediate outputs, ${z}^{h}\in {R}^{C\times H}$ and ${z}^{v}\in {R}^{C\times W}$.

To obtain the final output tensor $\text{Y}\in {R}^{C\times H\times W}$, which contains more informative global priors, ${z}^{h}$ and ${z}^{v}$ are combined as follows, resulting in a tensor $z\in {R}^{C\times H\times W}$, and the tensor $z$ can be described as:

16 $$\begin{array}{c}{z}_{c,m,n}={z}_{c,m}^{h}+{z}_{c,n}^{v}\end{array}$$

The output $\text{Y}$ can be described as:

17 $$\begin{array}{c}Y=Scale\left(x,\sigma \left(l\left(z\right)\right)\right)\end{array}$$

During the aggregation process, each position in the output tensor is connected to multiple positions in the input tensor. This is achieved through elemental multiplication ($Scale$), the Sigmoid function ($\sigma$), and a 1 × 1 convolution ($l$). In Fig. 7, the square in the output tensor is connected to all positions that share the same horizontal or vertical coordinates. By repeating this aggregation process iteratively, long-term dependencies can be established across the entire scene. Unlike global average pooling, strip pooling focuses on long but narrow ranges instead of the entire feature map. This approach avoids unnecessary connections between remote locations and highlights important areas at remote edge positions, effectively reducing the impact of distance changes on remote lane detection. Importantly, this method is theoretically feasible and has the potential to improve performance.

## Experiment

### Dataset

The experiments in this paper utilize two widely recognized benchmark datasets for lane detection: Tusimple dataset^33^ and CULane dataset^6^.

**Figure 8:**
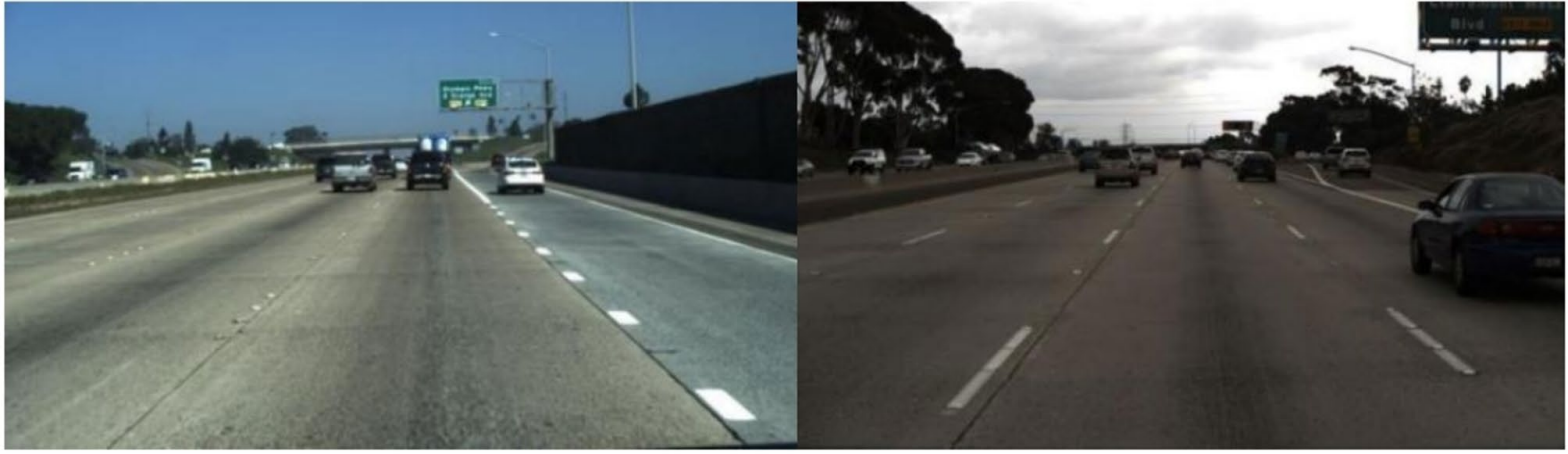
TuSimple dataset example.

TuSimple dataset is one of the large-scale datasets currently used for lane detection tasks, provided by Tucson Technology, including 3268 training images, 358 validation images, and 2782 test images. The resolution of the image is 1280 $\times$ 720 pixels. The filming scene is mainly on the highway, with mostly sunny weather conditions, sufficient lighting, and clear lane markings. Figure 8 shows some examples of images. Some of the images contain situations such as worn and blurred lane markings, vehicle occlusion, intersections, and high curvature curves, which play an important role in the comprehensive evaluation of the robustness and generalization ability of the algorithm.

CULane is a challenging and widely covered dataset of lane markings published in a paper in 2018. It effectively solves the problem of a single scene in the TuSimple dataset and can better measure the comprehensive performance of a lane detection algorithm. This dataset contains a total of 133,235 images, divided into three parts: training set, validation set, and test set. The three subsets contain 88,880 images, 9675 images, and 34,680 images, respectively.

**Figure 9:**
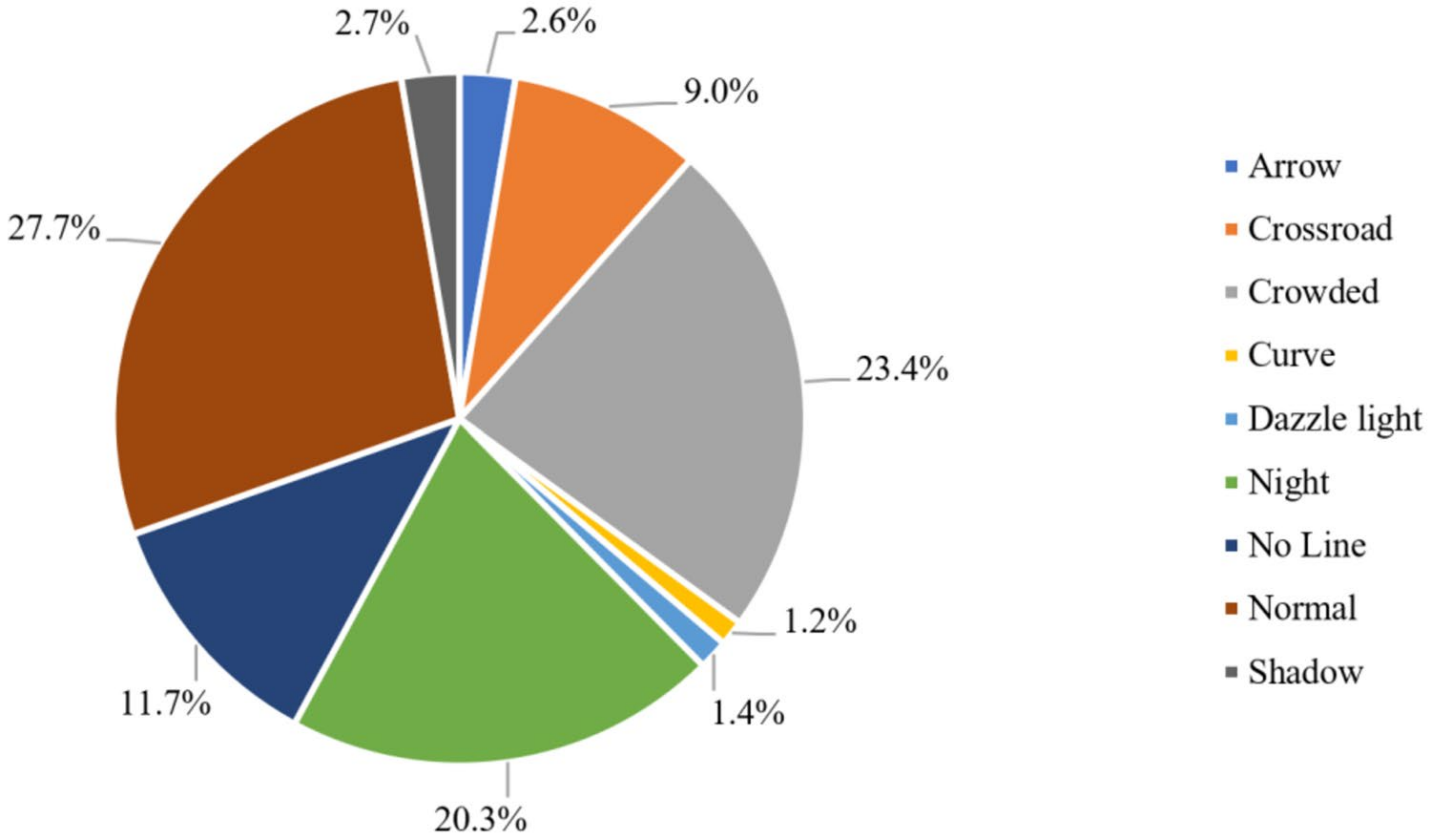
Scene distribution map of CULane dataset.

**Figure 10:**
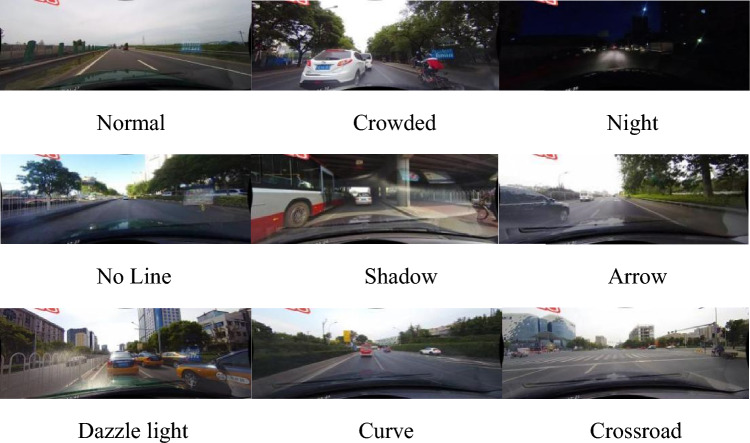
Example of CULane dataset.

CULane dataset contains nine different complex scenarios, as shown in Fig. 9, namely: Normal Crowded、Night、No line、Shadow、Arrow、Dazzle light、Curve、Crossroad. These images come from various scenes in Beijing and its surrounding rural areas, including urban roads, highways, etc., covering different times, weather conditions, lighting conditions, and other situations. Some example images of the CULane dataset are shown in Fig. 10. Table 1 in the paper provides additional details about these datasets.

**Table 1:** Datasets description.

Dataset	Train	Validation	Test	Resolution	Type	Lane
TuSimple	3268	358	2782	1280 $\times$ 720	Highway	$\le$ 5
CULane	88,880	9675	34,680	1640 $\times$ 590	Night, highway	$\le$ 4

For the TuSimple dataset, this paper uses accuracy as the main evaluation metric and evaluates it using independent evaluation source code. The definition of accuracy is as follows:

18 $$\begin{array}{c}Accuracy=\sum_{clip}\frac{{C}_{clip}}{{S}_{clip}}\end{array}$$

This evaluation metric measures the accuracy of predicting points on the lane line of a given image. It calculates the number of correctly predicted lane points and the total number of ground truth points in the given image. In addition, this paper also evaluated the false positive (FP) and false negative (FN) of the predicted results. The formulas for these indicators are as follows:

19 $$\begin{array}{c}FP=\frac{{F}_{pred}}{{N}_{pred}}\end{array}$$

20 $$\begin{array}{c}FN=\frac{{M}_{pred}}{{N}_{gt}}\end{array}$$

where ${F}_{pred}$ represents the number of incorrectly predicted lanes, ${N}_{pred}$ represents the total number of predicted lanes, ${M}_{pred}$ represents the number of predicted missed lanes, and ${N}_{gt}$ represents the actual number of lanes.

For the CULane dataset, the width of its lane lines is considered to be 30 pixels wide. When calculating the consistency between predicted and actual ground conditions, the Intersection over Union (IoU) criterion is used. The CULane dataset uses F1 score for performance evaluation, which takes into account both accuracy and recall, and is widely used in many classification problems. The F1 score is within the range of [0, 1], and the closer the score is to 1, the better the performance of the model. The specific calculation method is as follows:

21 $$\begin{array}{c}F1=\frac{2\times Precision\times Recall}{Precision+Recall}\end{array}$$

Precision refers to the proportion of samples that actually belong to the positive category predicted by the model, while Recall refers to the proportion of positive category samples predicted by the model to all true positive category samples. The IoU threshold is set to 0.5. True positive (TP) indicates a prediction with an IoU greater than 0.5, FP indicates a prediction with an IoU less than 0.5, true negative (TN) indicates that there is no such lane and no such lane is predicted, and FN indicates missed detection. There is such a lane but it is predicted that there is no such lane. The formulas for accuracy and recall are as follows:

22 $$\begin{array}{c}Precision=\frac{TP}{TP+FP}\end{array}$$

23 $$\begin{array}{c}Recall=\frac{TP}{TP+FN}\end{array}$$

### Implementation details

In this research, the original images from both Tusimple and CULane datasets are resized to 512 × 256 pixels and the RGB values are normalized from the range of 0–255 to 0 − 1. To enhance the dataset, various methods such as shading, noise addition, flipping, panning, rotating, and intensity change are applied. Additionally, to tackle the issue of data imbalance caused by a large number of image frames in both datasets, hard data with poor loss values are sampled during the training process to increase the selection ratio of challenging data.

The experiments are conducted using a GPU (RTX3060 TI 8 GB) and the code is implemented using PyTorch. During training, each batch consists of 2 images, and the learning rate is set to 0.0001 for Tusimple and 0.00001 for CULane in the optimization process. The training epoch for all datasets is set to 1500. The threshold and other hyperparameters are determined through experimentation.

The LHFFNet model achieves accurate lane line predictions by predicting the exact positions of lane points and fitting these points with spline curves.

### Comparison experiment

**Figure 11:**
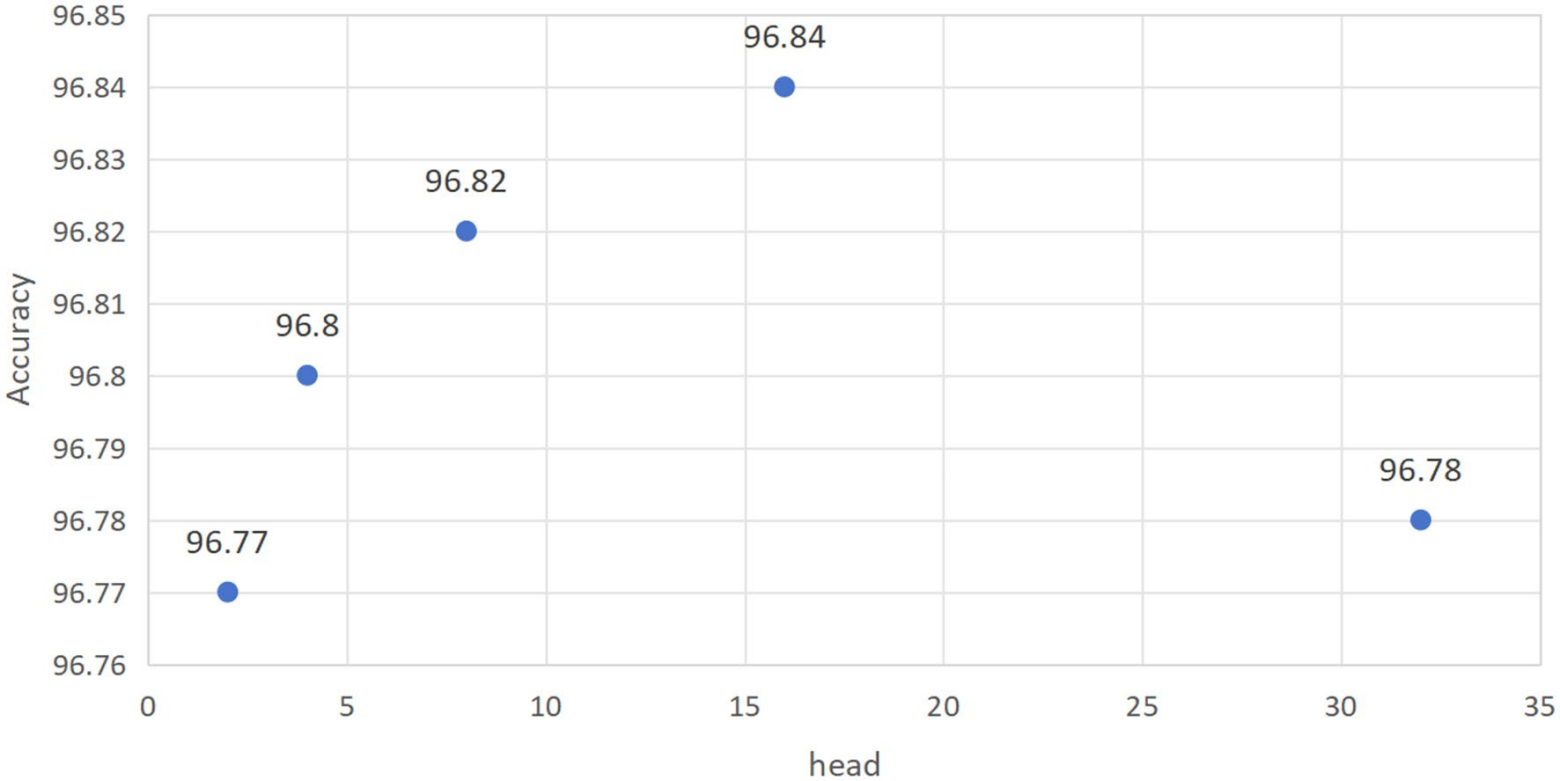
Comparison chart of accuracy results for setting the number of heads for MHSA in the Tusimple dataset, with the number of heads is set to 2, 4, 8, 16 and 32.

**Figure 12:**
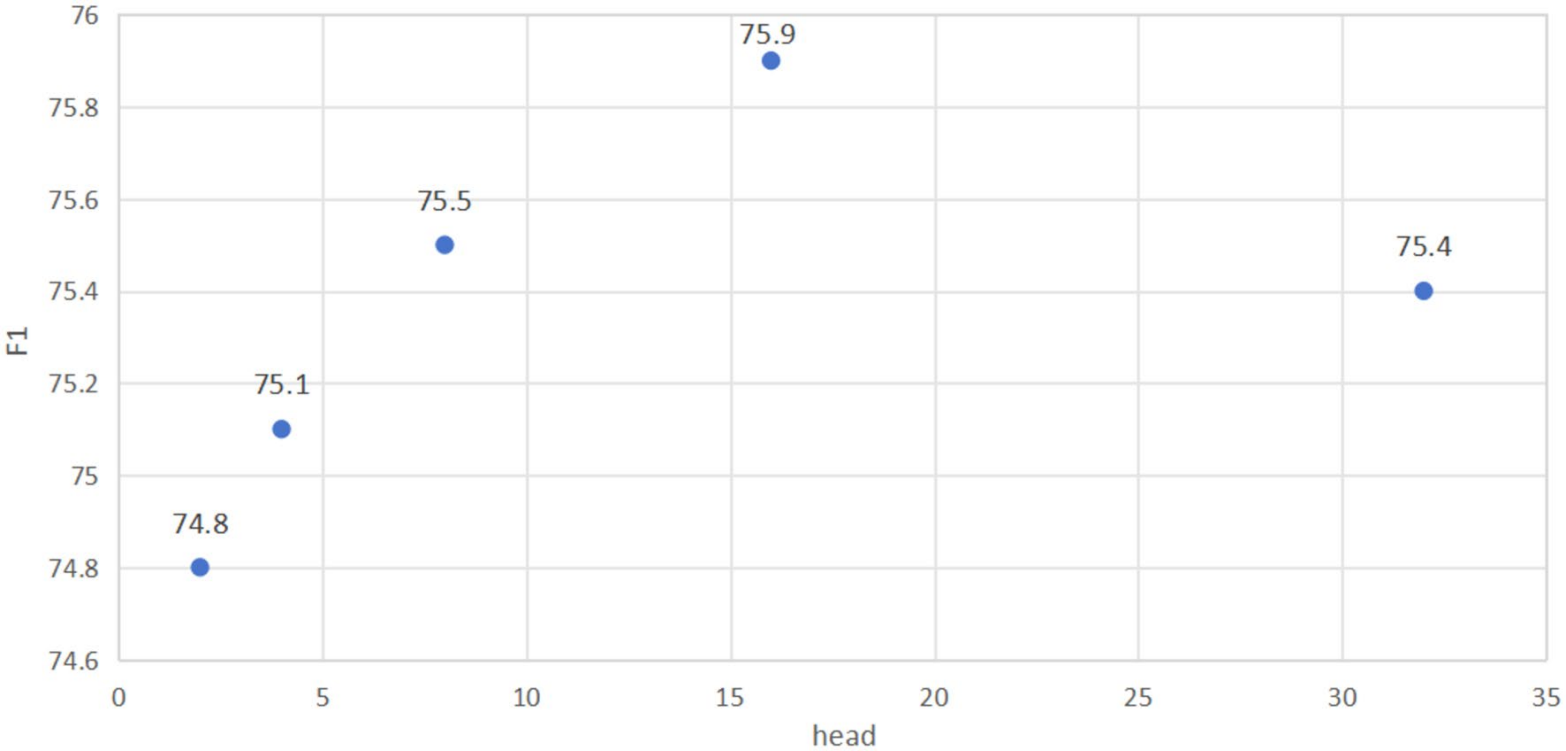
Comparison chart of F1 results for setting the number of heads for MHSA in the CULane dataset, with the number of heads is set to 2, 4, 8, 16 and 32.

In order to demonstrate the advantages of the proposed method in lane line detection task, this study compares the proposed method with other popular methods on different datasets. The experiments is conducted with SHN as the backbone and the experiments were labelled as LHFFNet. This paper explores the impact of the number of heads on lane detection performance of LHFFNet by setting the number of heads of MHSA in MAEM to 2, 4, 8, 16 and 32. Figure 11 and Fig. 12 respectively shows the impact of changes in the number of heads on the detection performance of LHFFNet in the Tusimple and CULane datasets. As the LHFFNet performs better when the number of heads is 8 and 16, the entire experiment will explore the effect of setting the number of heads to 8 and 16.

The paper presents the results of LHFFNet on TUsimple dataset and compares them with popular lane detection methods, including SCNN, RESA, PINet, Enet-SAD, LaneNet PolyLaneNet, UFLD, LaneATT and CondLaneNet^34^. As shown in Table 2, the evaluation of TuSimple dataset requires precise x-axis values with certain fixed y-axis values. LHFFNet shows high performance in term of accuracy and false positive rate. The false negative rate also shows reasonable value. LHFFNet(8) and LHFFNet(16) represents LHFFNet with 8 and 16 heads respectively in MHSA. LHFFNet(8) has lower FP and FN values when the detection accuracy is the same as RESA, suggesting that the method can detect lane lines more accurately. In particular, LHFFNet(16) achieves an advanced level of accuracy of 96.84% when the number of heads is set to 16. Compared to the PINet network, LHFFNet(16) achieved an accuracy improvement of nearly 0.1% on the TuSimple dataset. This is because the TuSimple dataset has a slightly simpler scene, and it is not difficult to see from the results of other models that most models can achieve detection accuracy of over 96%. Nevertheless, compared with other methods, LHFFNet still achieved an accuracy of 96.84%, and FP and FN decreased to 2.66% and 2.44%, respectively, which means that LHFFNet achieves higher accuracy on lane line detection task.

**Table 2:** Comparison of results with other models on the Tusimple dataset.

Method	Accuracy (%)	FP (%)	FN (%)
SCNN	96.53	6.17	1.80
RESA	96.82	3.63	2.48
PINet(4H)	96.75	3.10	2.50
ENet-SAD	96.64	6.02	2.05
LaneNet	96.38	7.80	2.44
PolyLaneNet	93.36	9.42	9.33
UFLD	95.86	18.91	3.75
LaneATT	95.63	3.53	2.92
CondLaneNet	96.54	2.01	3.50
LHFFNet(8)	96.82	2.43	2.36
LHFFNet(16)	96.84	2.66	2.44

**Figure 13:**
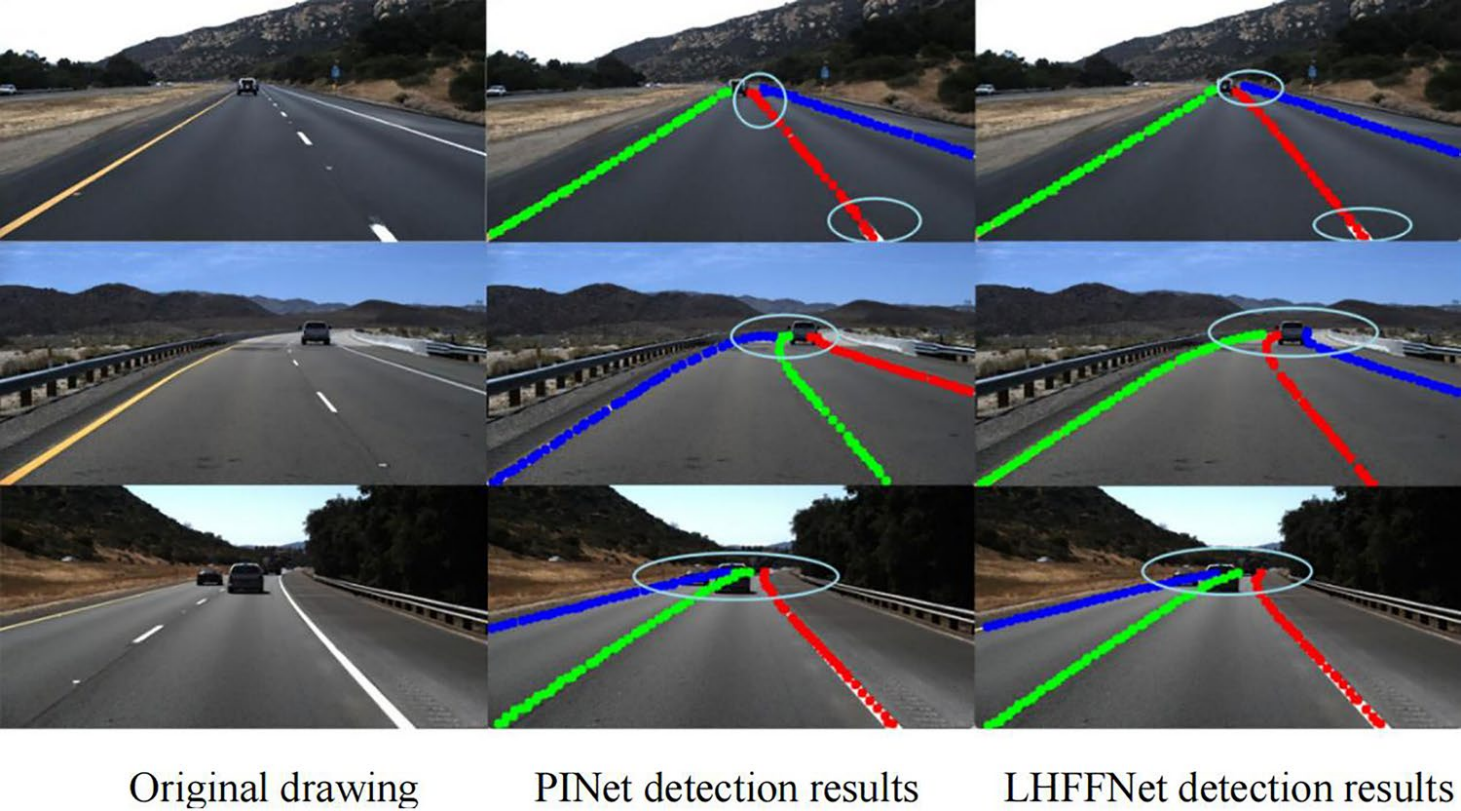
Detection Results of TuSimple Dataset.

Figure 13 shows a comparison of the specific detection results of the proposed model LHFFNet on the TuSimple dataset with PINet.

In the case of simple road scenes, fewer lanes, and obvious lane line features, it can be seen that LHFFNet and PINet have achieved relatively accurate detection results for lane lines, and both models have good detection results. However, PINet may experience slight deviation in the predicted lane lines at the near and far ends, especially when the features at the far end are not obvious and there is vehicle occlusion, resulting in significant deviation and point accumulation in its detection results. LHFFNet achieves more accurate detection at both the far and near ends, and the predicted points can perfectly cover the real lane line. Whether at the near or far end, The detection results of LHFFNet greatly reduce deviation and point accumulation. In curve detection with vehicle occlusion at the far end, the fitting effect of LHFFNet on the lane line is also smoother.

This paper conducts comparative experiments on representative algorithms in recent years on CULane, mainly comparing algorithms such as SCNN, LaneATT, RESA, PINet, UFLD, and LaneAF^35^. The specific results in each scenario are shown in Table 3. Comparing the results of LHFFNet with other models when the number of heads is 16, it can be seen from Table 3 that the F1 score of LHFFNet(16) is 75.9%, which exceeds some previous lane detection methods. In the Crowd and No Line scenarios, the best results were achieved compared with other models. In various scenarios, compared to PINet, all detection results have improved, achieving a growth of 3.5% in the Shadow scenario, 4.1% and 4.4% in the Arrow and Curve scenarios, and 2.9% in the Night scenario. In other scenarios (except for Cross), the detection results have improved by more than 1% compared to PINet. LHFFNet has achieved performance improvement compared to PINet, and its detection performance is also highly competitive compared to other mainstream models.

**Table 3:** Comparison of results with other models on the CULane dataset (Show only FP for cross).

	SCNN	LaneATT	RESA	PINet	UFLD	LaneAF	LHFFNet(8)	LHFFNet(16)
Normal	90.6	91.2	92.1	90.3	90.7	91.1	91.5	92.0
Crowd	69.7	72.8	73.1	72.3	70.2	73.3	72.8	73.4
Dazzle	58.5	65.8	69.2	66.3	59.5	69.7	65.9	67.3
Shadow	66.9	68.0	72.8	68.4	69.3	75.8	70.7	71.9
No Line	43.4	49.1	47.7	49.8	44.4	50.6	50.1	51.3
Arrow	84.1	87.8	88.3	83.7	85.7	86.9	88.0	87.8
Curve	64.4	63.8	70.3	65.2	69.5	65.0	67.0	69.6
Cross	1990	1020	1503	1427	2037	1844	1397	1401
Night	66.1	68.6	69.9	67.7	66.7	70.9	71.7	70.6
F1	71.6	75.1	75.3	74.4	72.3	75.6	75.5	75.9

**Figure 14:**
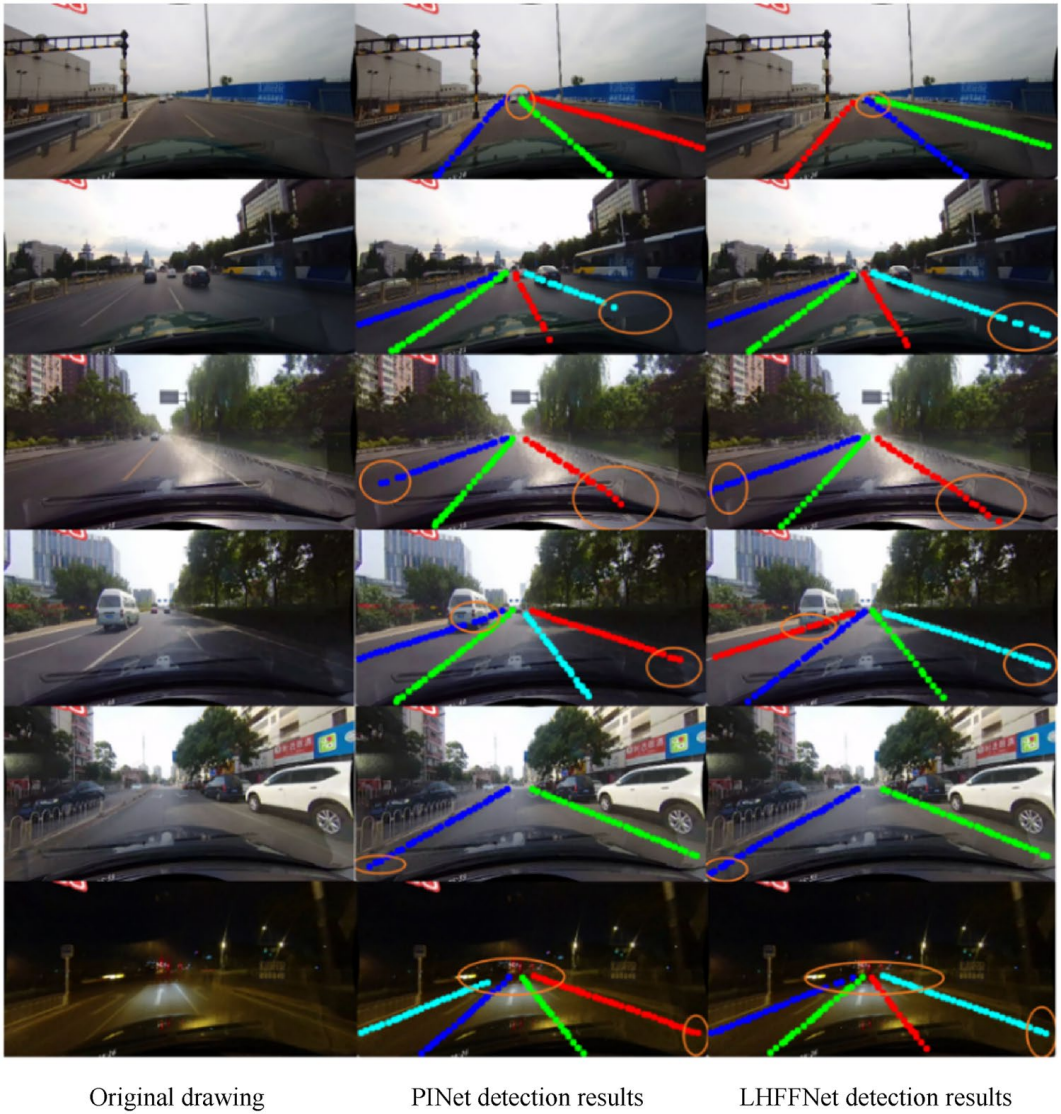
Detection Results of CULane Dataset.

Figure 14 shows a comparison of the detection results of LHFFNet in multiple scenes in CULane, From top to bottom, they are Normal, No line, Dazzle light, Shadow, Arrow and Night. LHFFNet can achieve good detection results for these complex road scenes. From the Fig. 14, it can be seen that LHFFNet fits the lane lines well in various scenarios, and in each scenario, the lane lines fitted by LHFFNet are farther away and have a smoother shape compared to those fitted by PINet. For areas that PINet did not predict, LHFFNet predicted lane lines with slightly sparse key point arrangements, demonstrating the superiority of mixed feature fusion of LHFFNet in lane line modeling.

### Ablation experiment

The effectiveness of MAEM, SCDM and SPM are discussed in detail in this paper, the advantages of each module are analyzed in detail. In order to verify the effectiveness of these different components, experiments are carried out on Tusimiple dataset and CULane dataset to demonstrate their performance. LHFFNet chooses SHN as the backbone of extracting lane feature information. Meanwhile, in this paper, the ablation experiment was discussed when the number of heads in MAEM was set to 8 and 16. Tables 4 and 5 show the results of ablation in TuSimple, and Tables 6 and 7 show the results of ablation in CULane. The experiment fully proves the influence of different number of heads and each module on the baseline, and confirms the ability of the proposed module. The ablation study uses official indicators to calculate the average performance gap.

**Table 4:** The number of heads for MHSA is set to 8, and the study of modular ablation is performed on Tusimple dataset, with the baseline representing the results without any substitution or introduction operations.

Baseline	MAEM	SCDM	SPM	Accuracy(%)	FP(%)	FN(%)
√				96.75	3.10	2.50
√	√			96.77	3.26	2.49
√		√		96.73	3.15	2.63
√			√	96.76	3.16	2.53
√	√	√		96.78	3.02	2.61
√	√		√	96.79	2.67	2.52
√		√	√	96.76	2.90	2.56
√	√	√	√	96.82	2.43	2.36

**Table 5:** The number of heads for MHSA is set to 16, and the study of modular ablation is performed on Tusimple dataset, with the baseline representing the results without any substitution or introduction operations.

Baseline	MAEM	SCDM	SPM	Accuracy(%)	FP(%)	FN(%)
√				96.75	3.10	2.50
√	√			96.77	3.18	2.54
√		√		96.74	2.96	2.56
√			√	96.76	3.11	2.60
√	√	√		96.76	2.90	2.49
√	√		√	96.80	2.52	2.51
√		√	√	96.77	2.70	2.47
√	√	√	√	96.84	2.66	2.44

**Table 6:** The number of heads for MHSA is set to 8, and the study of modular ablation is performed on CULane dataset, with the baseline representing the results without any substitution or introduction operations.

Baseline	MAEM	SCDM	SPM	F1 (%)
√				74.4
√	√			74.9
√	√	√		75.2
√	√	√	√	75.5

**Table 7:** The number of heads for MHSA is set to 16, and the study of modular ablation is performed on CULane dataset, with the baseline representing the results without any substitution or introduction operations.

Baseline	MAEM	SCDM	SPM	F1 (%)
√				74.4
√	√			75.1
√	√	√		75.3
√	√	√	√	75.9

Explore with the number of heads set to 8. In the TuSimple dataset, after adding the MAEM module to the baseline network, the accuracy improved to 96.77%. At the same time, adding MAEM and SCDM resulted in a small improvement in accuracy, and the FP decreased compared to the baseline, which means that the overall detection performance of the model is more stable. Simultaneously adding MAEM and SPM resulted in an accuracy of 96.79%, with a slight increase in FN. After all three modules were added to PINet, the accuracy was 96.82%, FP was 2.43%, and FN reached the lowest level compared to before, at 2.36%. In the CULane dataset, the baseline network increased F1 to 74.9% after adding MAEM and 75.2% after adding SCDM. Finally, with the addition of SPM, the F1 score increased to 75.5%. Although adding SCDM brings an increase in the number of parameters to the model, it does not affect the real-time detection speed of the model.

Then explore with the number of heads set to 16. In the TuSimple dataset, after adding the MAEM module to the baseline network, the accuracy improved to 96.77%. At the same time, adding MAEM and SPM, the accuracy improved to 96.80%, FP decreased to 2.52%, FN was 2.51%. Then, after adding SCDM, the accuracy reached 96.84%, compared to joining MAEM and SPM, FP slightly increased, FN slightly decreased, and overall remained stable. Analysis of the CULane dataset results showed that the baseline network increased F1 to 75.1% after adding MAEM, then to 75.3% after adding SCDM, and finally to 75.9% after adding SPM. The experimental results show that regardless of whether the number of heads is 8 or 16, with the subsequent addition of modules, the accuracy of TuSimple and the F1 score of CULane overall increase, indicating that each module is effective in improving model performance.

## Conclusion

This paper proposes a multi-scale and multi-space feature enhancement fusion method for lane detection, LHFFNet. This method designs a MAEM that combines MHSA to enhance the correlation of multi-scale lane features, utilize multi head self attention for multi-space attention enhancement, and achieves multi-space feature enhancement to enhance output logic for subsequent feature fusion. SCDM introduces spatial separable convolutional branches to connect multi-scale lane line features, retain different scale feature information, and emphasize important lane areas in multi-scale feature information in feature maps with variable spatial distribution. SPM flexibly refines lane features at edge positions (especially at the far end), obtaining more accurate contextual information and facilitating accurate modeling of subsequent lane lines. LHFFNet further improves the detection performance of the original model for lane markings, which is of great significance for the safety of autonomous driving. However, for heavy rain and muddy roads, the detection performance of the model in lane markings still needs to be enhanced. The next step of work will focus on addressing this defect, constructing a dataset for rainy driving, and conducting corresponding model construction and testing.

## Discussion

The discussion must not contain subheadings.

## Data Availability

The current datasets used are the open-source datasets TuSimple (https://github.com/TuSimple) and CULane (https://xingangpan.github.io/projects/CULane.html). For specific usage procedures of the data and the analyzed data can be obtained from the corresponding author.

## References

[1] Li X, Li J, Hu X, Yang J (2019). Line-cnn: End-to-end traffic line detection with line proposal unit. IEEE Trans. Intell. Transp. Syst..

[2] He Y, Wang H, Zhang B (2004). Color-based road detection in urban traffic scenes. IEEE Trans. Intell. Transp. Syst..

[3] Chiu KY, Lin SF (2005). Lane detection using color-based segmentation. IEEE Proc. Intell. Vehicles Sympos..

[4] Wang Y, Shen D, Teoh EK (1998). Lane detection using catmull-rom spline. In IEEE Int. Conf. Intell.Veh..

[5] Lee C, Moon JH (2018). Robust lane detection and tracking for real-time applications. IEEE Trans. Intell. Transp. Syst..

[6] Pan, X., Shi, J., Luo, P., Wang, X., & Tang, X. Spatial as deep: Spatial cnn for traffic scene understanding. *Proc. AAAI conference on Artif. Intell*. Vol. 32, No. 1 (2018).

[7] Qin Z, Wang H, Li X, Vedaldi A (2020). Ultra fast structure-aware deep lane detection. Computer Vision–ECCV 2020: 16th European Conference Proceedings.

[8] Philion J (2019). Fastdraw: Addressing the long tail of lane detection by adapting a sequential prediction network. Proc. IEEE Conf. Comput. Vis. Pattern Recogn..

[9] Chen, Z., Liu, Q., & Lian, C. Pointlanenet: Efficient end-to-end cnns for accurate real-time lane detection. In *2019 IEEE intelligent vehicles symposium (IV)*. 2563–2568 (2019).

[10] Tabelini L, Berriel R, Paixao TM, Badue C, De Souza AF, Oliveira-Santos T (2021). Keep your eyes on the lane: Real-time attention-guided lane detection. Proc. IEEE Conf. Comput. Vis. Pattern Recogn..

[11] Tabelini, L., Berriel, R., Paixao, T. M., Badue, C., De Souza, A. F., & Oliveira-Santos, T. Polylanenet: Lane estimation via deep polynomial regression. In *2020 25th International Conference on Pattern Recognition (ICPR)*. 6150–6156 (2021).

[12] Bi, Q., Qin, K., Li, Z., Zhang, H., & Xu, K. Multiple instance dense connected convolution neural network for aerial image scene classification. In *2019 IEEE International Conference on Image Processing (ICIP)*. 2019, 2501–2505 (2019).

[13] Cheng G, Yang C, Yao X, Guo L, Han J (2018). When deep learning meets metric learning: Remote sensing image scene classification via learning discriminative CNNs. IEEE Trans. Geosci. Remote Sens..

[14] Lin TY, Dollár P, Girshick R, He K, Hariharan B, Belongie S (2017). Feature pyramid networks for object detection. Proc. IEEE Conf. Comput. Vis. Pattern Recogn..

[15] Newell A, Yang K, Deng J, Leibe B (2016). Stacked hourglass networks for human pose estimation. Computer Vision–ECCV 2016: 14th European Conference, Amsterdam, The Netherlands, Proceedings.

[16] Ko Y, Lee Y, Azam S, Munir F, Jeon M, Pedrycz W (2021). Key points estimation and point instance segmentation approach for lane detection. IEEE Trans. Intell. Transp. Syst..

[17] Vaswani A, Shazeer N, Parmar N, Uszkoreit J, Jones L, Gomez AN, Polosukhin I (2017). Attention is all you need. Adv. Neural. Inf. Process. Syst..

[18] Hou Q, Zhang L, Cheng MM, Feng J (2020). Strip pooling: Rethinking spatial pooling for scene parsing. Proc. IEEE Conf. Comput. Vis. Pattern Recogn..

[19] Andrade DC, Bueno F, Franco FR, Silva RA, Neme JHZ, Margraf E, Dos Santos Amaral R (2018). A novel strategy for road lane detection and tracking based on a vehicle’s forward monocular camera. IEEE Trans. Intell. Trans. Syst..

[20] Alvarez JM, López AM, Gevers T, Lumbreras F (2014). Combining priors, appearance, and context for road detection. IEEE Trans. Intell. Transp. Syst..

[21] Zheng T, Fang H, Zhang Y, Tang W, Yang Z, Liu H, Cai D (2021). Resa: Recurrent feature-shift aggregator for lane detection. Proc. AAAI Conf. Artif. Intell..

[22] Hou Y, Ma Z, Liu C, Loy CC (2019). Learning lightweight lane detection cnns by self attention distillation. Proc. IEEE/CVF Int.Conf. Comput. Vis..

[23] Neven D, De Brabandere B, Georgoulis S, Proesmans M, Van Gool L (2018). 2018 Towards end-to-end lane detection: An instance segmentation approach. IEEE Intell. Vehicles Sympos. (IV)..

[24] Qu Z, Jin H, Zhou Y, Yang Z, Zhang W (2021). Focus on local: Detecting lane marker from bottom up via key point. Proc. IEEE Conf. Comput. Vis. Pattern Recogn..

[25] Liu R, Yuan Z, Liu T, Xiong Z (2021). End-to-end lane shape prediction with transformers. Proc. IEEE/CVF Int.Conf. Comput. Vis..

[26] Jaderberg, M., Simonyan, K. & Zisserman, A. Spatial transformer networks. *Advances in neural information processing systems 2015*, Vol. 28 (2015).

[27] Zhang H, Goodfellow I, Metaxas D, Odena A (2019). Self-attention generative adversarial networks. Int. Conf. Mach. Learn..

[28] Wang X, Girshick R, Gupta A, He K (2018). Non-local neural networks. Proc. IEEE Conf. Comput. Vis. Pattern Recogn..

[29] Srinivas A, Lin TY, Parmar N, Shlens J, Abbeel P, Vaswani A (2021). Bottleneck transformers for visual recognition. Proc. IEEE/CVF Conf. Comput. Vis. Pattern Recogn..

[30] Shaw, P., Uszkoreit, J., & Vaswani, A. Self-attention with relative position representations. *arXiv preprint *arXiv:1803.02155 (2018).

[31] Zhao H, Jia J, Koltun V (2020). Exploring self-attention for image recognition. Proc. IEEE/CVF Conf. Comput. Vis. Pattern Recogn..

[32] Bello, I., Zoph, B., Vaswani, A., Shlens, J. & Le, Q. V. Attention augmented convolutional networks. In *Proceedings of the IEEE/CVF international conference on computer vision 2019*. 3286–3295 (2019).

[33] Tusimple benchmark. In: Available Online: https://github.com/TuSimple/ tusimple-benchmark (2019).

[34] Liu L, Chen X, Zhu S, Tan P (2021). Condlanenet: a top-to-down lane detection framework based on conditional convolution. Proc. IEEE/CVF Int.Conf. Comput. Vis..

[35] Abualsaud H, Liu S, Lu DB, Situ K, Rangesh A, Trivedi MM (2021). Laneaf: Robust multi-lane detection with affinity fields. IEEE Robot. Autom. Lett..

